# Planarian Body-Wall Muscle: Regeneration and Function beyond a Simple Skeletal Support

**DOI:** 10.3389/fcell.2016.00008

**Published:** 2016-02-08

**Authors:** Francesc Cebrià

**Affiliations:** Department of Genetics, Faculty of Biology, Institute of Biomedicine of the University of Barcelona, University of BarcelonaBarcelona, Spain

**Keywords:** planarian, myosin heavy chain, MyoD, regeneration, stem cells, myogenesis, myocytes, positional information

## Abstract

The body-wall musculature of adult planarians consists of intricately organized muscle fibers, which after amputation are regenerated rapidly and with great precision through the proliferation and differentiation of pluripotent stem cells. These traits make the planarian body-wall musculature a potentially useful model for the study of cell proliferation, differentiation, and pattern formation. Planarian body-wall muscle shows some ambiguous features common to both skeletal and smooth muscle cells. However, its skeletal nature is implied by the expression of skeletal myosin heavy-chain genes and the myogenic transcription factor *myoD*. Where and when planarian stem cells become committed to the myogenic lineage during regeneration, how the new muscle cells are integrated into the pre-existing muscle net, and the identity of the molecular pathway controlling the myogenic gene program are key aspects of planarian muscle regeneration that need to be addressed. Expression of the conserved transcription factor *myoD* has been recently demonstrated in putative myogenic progenitors. Moreover, recent studies suggest that differentiated muscle cells may provide positional information to planarian stem cells during regeneration. Here, I review the limited available knowledge on planarian muscle regeneration.

## Introduction

In the last 10–15 years stem cell-based regenerative medicine has emerged as a vigorous research field within the biological sciences (Atala et al., 2011). The obvious long-term goal is to develop treatments for diseases and traumatic injuries for which no cure is currently available. One of the consequences of growing interest in regenerative medicine is that the scientific community has refocused its attention on animal models capable of regenerating different cell types, tissues, organs, and structures under natural conditions. Vertebrates in general, and mammals in particular, have very limited regenerative capabilities. However, compared with other tissues, mammalian skeletal muscle shows a significant degree of repair and regeneration. This capacity is conferred by muscle stem cells, known as satellite cells, which are usually quiescent but can be activated in response to injury or stress to proliferate and give rise to muscle progenitors that differentiate into new muscle tissue (see Dumont et al., 2015 for a recent review). Quiescent satellite cells express the gene *Pax7* and have been shown in several studies to be essential for skeletal muscle regeneration (Lepper et al., 2011; Sambasivan et al., 2011). Among vertebrates, salamanders are well known for their limb regenerating abilities. Remarkably, a recent study reported that 2 different species of salamanders use different strategies to regenerate their skeletal muscle (Sandoval-Guzmán et al., 2014): while *Pax7*-positive satellite cells are the main source of regenerated muscle in axolotls, most new muscle fibers in newts are derived from the dedifferentiation of pre-existing muscle cells that re-enter the cell cycle to give rise to new muscle cells.

Among the several regeneration models commonly used, freshwater planarians are unique in that (i) they can regenerate an entire animal from a tiny portion of the body; and (ii) can do so thanks to the presence of a population of adult somatic pluripotent stem cells (Reddien and Sánchez Alvarado, 2004; Baguñà, 2012; Rink, 2013). These animals are thus an attractive model for the *in vivo* study of the behavior of totipotent stem cells (Gentile et al., 2011). Here, I review the rather scarce current knowledge of planarian muscle and its regeneration and report on the existing tools used to study how planarian stem cells are regulated *in vivo* to give rise to new muscle cells during regeneration and daily cell turnover. Moreover, I discuss recent data suggesting that planarian muscle fibers, in addition to providing skeletal support, may play a key role in providing positional information to stem cells so they differentiate into the correct cell types and tissues.

## Planarian musculature

Platyhelminthes are acoelomate, triploblastic, bilaterally symmetrical animals that lack circulatory, skeletal, and respiratory systems. Their bodies are surrounded by a dense and compact net of subepidermal muscle fibers arranged in different orientations. Locomotion in these planarians basically occurs through ciliary gliding. Muscles may support this locomotion and are mainly used to orientate the direction of the movement. Moreover, the muscle network acts against the hydrostatic skeleton consisting of the fluids of the gut, parenchymal cells, and other organs (Clark, 1964; Rieger et al., 1994). The musculature thus mainly serves to maintain the shape and integrity of the body. In addition to the body-wall musculature, Platyhelminthes possess muscle fibers around the digestive system, reproductive organs, and mouth opening, and within the pharynx.

The body-wall musculature of Platyhelminthes consists of a variable number of layers of muscle fibers lying in different orientations, and its structure varies depending on body size (for reviews see Rieger et al., 1991; Hooge, 2001). Small Platyhelminthes such as acoels and catenulids have a simple body-wall musculature, consisting of an outer layer of circular fibers and an internal layer of longitudinal fibers (Crezée, 1975; Moraczewski, 1981). Larger Platyhelminthes possess a thicker musculature, in many cases accompanied by a layer of diagonal fibers between the outer and inner muscles (Rieger et al., 1994). On the other hand, the body-wall musculature of most polyclads consists of up to 5 or 6 layers of fibers (Prudhoe, 1985). The origin of the diagonal muscle fibers is not entirely clear: whereas Westblad (1949) proposed that these fibers are produced by the longitudinal musculature, Riser (1987) maintained that they are derived from circular muscle. According to Clark (1964) the diagonal fibers may act to flatten the body in larger platyhelminth species with very extensible bodies (e.g., triclads). In general, the body-wall musculature of the ventral aspect is more developed than that of the dorsal aspect.

**Figure 1:**
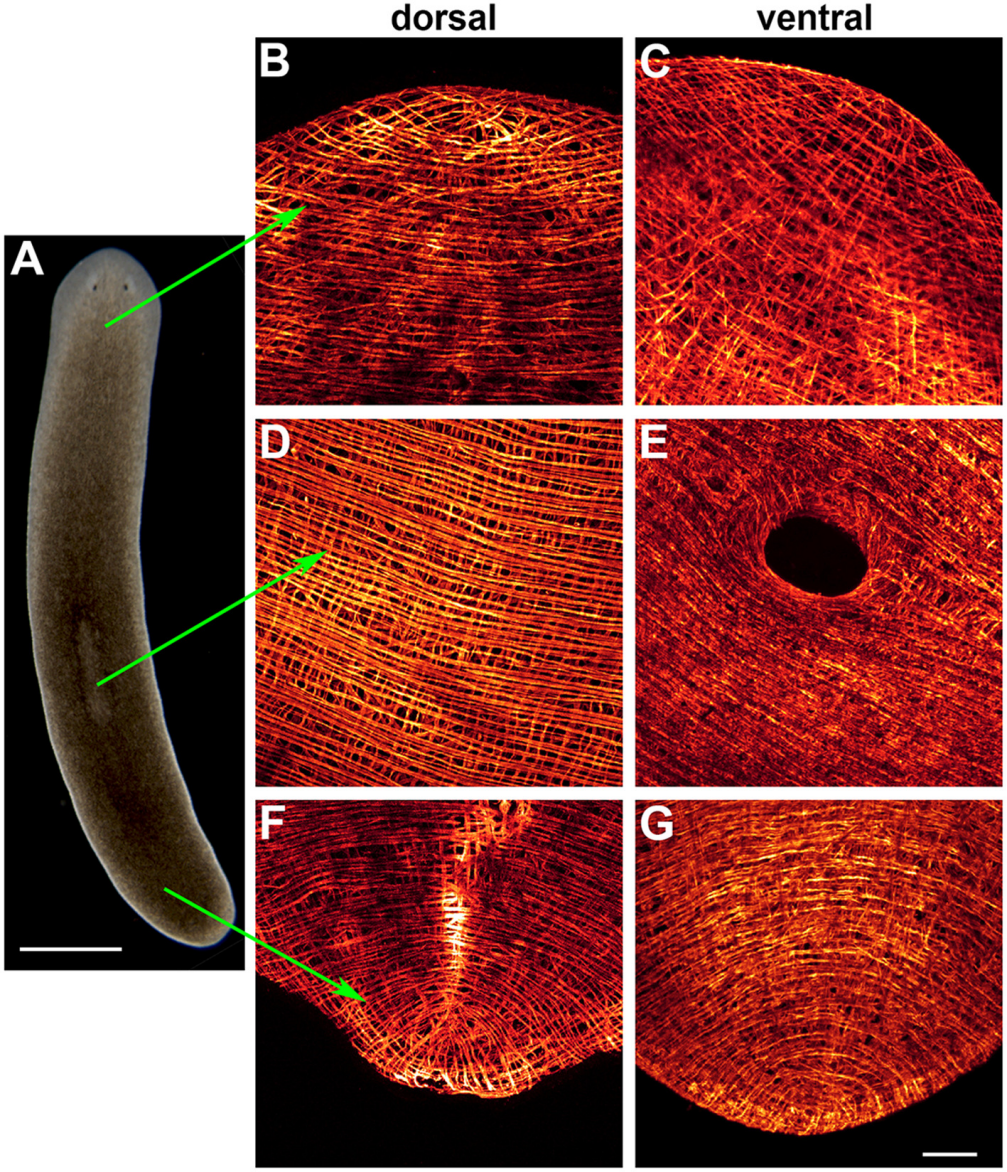
**Body-wall musculature of ***Schmidtea mediterranea***. (A)** Live animal. **(B–G)** Whole-mount immunostaining with TMUS-13 antibody, which recognizes the myosin heavy-chain (MHC) protein. The hole in **(E)** corresponds to the mouth opening. Scale bar: 1 mm for **(A)** and 50 μm for **(B–G)**. Image adapted from Cebrià (2000).

The body-wall musculature of the freshwater planarians *Dugesia trigrina* and *Schmidtea mediterranea* consists of 4 layers of fibers: circular, longitudinal, diagonal, and longitudinal fibers (from outside to inside). The inner longitudinal fibers are thicker than the outer ones. These layers are compressed within a region of 7–12 μm thick below the epidermis (Cebrià et al., 1997; Cebrià, 2000). In addition, a large number of dorsoventral fibers connect dorsal and ventral body surfaces. These fibers are more abundant in the tips and margins of the animal than in the central region of the body. All these fibers are arranged to form a dense, compact muscle net (Cebrià et al., 1997; Figure 1). The pattern of the inner longitudinal fibers differs between the dorsal and ventral surfaces of the anterior tip of the animal; dorsally, these fibers appear to converge toward a central zone at the anterior tip, whereas the ventral fibers run in parallel or even diverge in a fan-shaped pattern as they approach this tip (Figure 1).

## Planarian myosin heavy-chain genes

**Figure 2:**
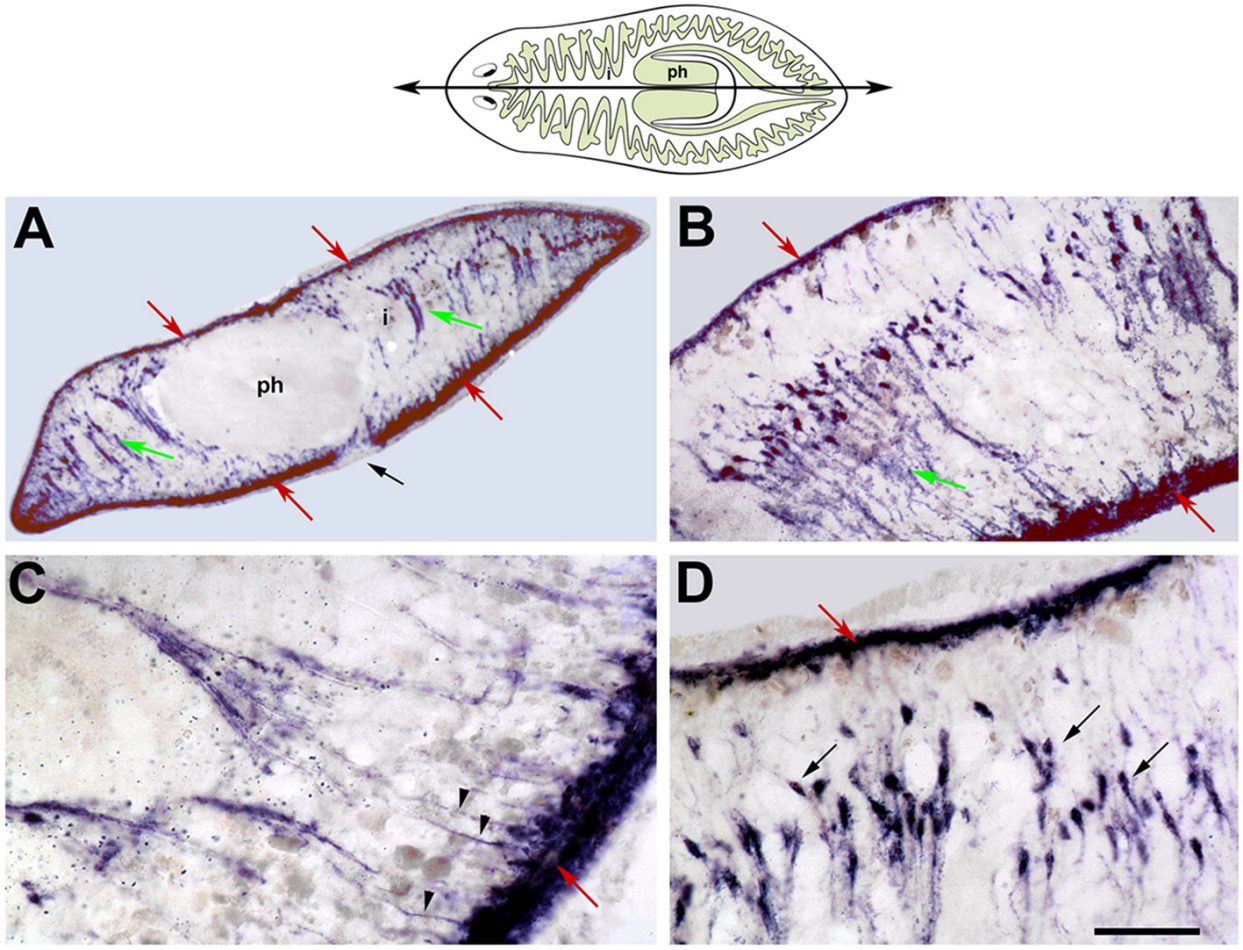
*****SmedmhcA*** expression in intact planarians**. *In situ* hybridization on sagittal histological sections. The cartoon represents the level and orientiation of the sagittal sections in **(A–D)**. The pharynx (ph) and the higly branched intestine (i) are highlighted. **(A)** The *SmedmhcA* gene is expressed in the body-wall musculature and dorsoventral fibers. No expression is detected in the pharynx (ph), around the intestinal ducts (i), or around the mouth opening (black arrow). **(B)** Higher magnification images reveal that ventral body-wall muscle is more developed than the corresponding dorsal muscle. **(C)** The dorsoventral muscle fibers attach to the body-wall surface as individual myofibers (arrowheads). **(D)** The nuclei of the dorsoventral muscle fibers (black arrows) are aligned and closer to the dorsal surface. Red arrows point to body-wall musculature. Green arrows poin tto dorsoventral fibers. In all figures, the anterior end is oriented to the left and the dorsal side to the top. Scale bars: **(A)**, 200 μm; **(B)**, 100 μm; **(C,D)**, 50 μm. Image adapted from Cebrià (2000).

Myosin proteins are highly conserved in all eukaryotic cells, in which they provide the motor force necessary for different kinds of movements, including cytokinesis, phagocytosis, organelle movement, and muscle contraction (Hartman and Spudich, 2012). Among the different types of myosins, myosin II proteins include those involved in muscle cell contraction. These consist of 2 heavy and 4 light chains. Two different myosin heavy-chain (*mhc*) genes encoding 2 different muscle fiber types have been identified in freshwater planarians. One is expressed in the muscle fibers of the pharynx, the muscles surrounding the gastrodermis, in a few scattered cells throughout the body-wall, and in some muscle fibers in the mesenchyme at the base of the pharynx. The other *mhc* gene is expressed in the subepidermal body-wall musculature and in the dorsoventral fibers (Kobayashi et al., 1998; Cebrià et al., 1999; Cebrià, 2000; Orii et al., 2002; Figure 2). The MHC protein possesses ATPase activity which provides the energy required for muscle contraction. Since the contraction velocity and ATPase activity of a muscle fiber can vary depending on its *mhc* isoform composition (Bárány, 1967), it is possible that the different expression patterns of planarian *mhc* genes confer different physiological properties to planarian muscle fibers. Accordingly, each MHC isoform may mediate different biological functions, such as locomotion (body-wall muscle) or peristaltic movements during food intake (pharynx and enteric muscle).

Vertebrates possess 3 main types of muscle: skeletal, cardiac, and smooth. Most invertebrates also have striated and smooth muscles, and in some cases, an oblique musculature with intermediate features. The muscle of planarians (and other Platyhelminthes) exhibits several ambiguities: like vertebrate smooth muscle cells planarian muscle cells are mononucleated, and can range from 150–200 μm in length and 5–10 μm in width (MacRae, 1963; Baguñà and Romero, 1981). At the physiological level, the lack of inhibition of ATPase activity at low pH values shown by *Girardia tigrina* muscle is a feature typical of the mature smooth fibers and embryonic skeletal fibers of vertebrates (Sarnat, 1984). At the ultrastructural level, the muscle myofilaments of many Platyhelminthes are arranged in a configuration typical of vertebrate smooth muscle, with dense bodies irregularly distributed (Rieger et al., 1991). In some cases however, these dense bodies are substituted by other structures known as Z bars, which are also irregularly distributed (MacRae, 1963, 1965; Morita, 1965; Reuter, 1977; Hori, 1983; Ehlers, 1985). These Z bar-containing muscles can in fact be considered obliquely striated (Lanzavecchia, 1977; Ehlers, 1985). Moreover, electron microscopy analyses of muscles with dense bodies reveal an oblique alignment (MacRae, 1965; Rieger and Mainitz, 1977).

Although planarian *mhc* genes are expressed in different muscle types with different physiological functions, phylogenetic analyses indicate that they are more similar to *mhc* genes in striated muscle from other animals, including vertebrates, than to smooth muscle-type *mhc* genes (Kobayashi et al., 1998; Cebrià, 2000).

## Planarian muscle regeneration

Freshwater planarians are mainly known for their extraordinary regenerative capabilities. These animals can regenerate an entire organism, including a *de novo* central nervous system, from a tiny piece of their bodies in only a few days (Newmark and Sánchez Alvarado, 2002; Reddien and Sánchez Alvarado, 2004; Cebrià et al., 2010). These abilities are conferred by the presence of a unique population of pluripotent adult stem cells called neoblasts (Newmark and Sánchez Alvarado, 2000; Baguñà, 2012; Rink, 2013; Adell et al., 2014). Upon amputation, neoblasts around the wound proliferate and give rise to the regenerative blastema, in which they differentiate into all the cell types required to restore the missing structures. Thus, unlike other organisms in which muscle regeneration depends on either the reactivation of unipotent stem cells (i.e., satellite cells) or the dedifferentiation of preexisting muscle cells that then re-enter the cell cycle to produce an expanded population of new muscle cells and fibers (Lepper et al., 2011; Sambasivan et al., 2011; Sandoval-Guzmán et al., 2014), new muscle cells in planarians arise from pluripotent neoblasts.

### Planarian neoblasts and muscle progenitors

Neoblasts are the only dividing cells in planarians and are basically defined by morphological criteria (small round cells of 5–10 μm of diameter with a very large nucleus and a scant cytoplasm) and by the expression of genes and proteins associated with cell division including histone H2B, PCNA, phosphohistone H3, and *Smedwi-1* (a *piwi* gene homolog; Reddien et al., 2005a). However, recent studies have shown that neoblasts are in fact a heterogeneous cell population consisting of truly pluripotent stem cells, the c-neoblasts (Wagner et al., 2011) as well as distinct subpopulations of lineage-committed progenitor cells (Scimone et al., 2014). These progenitors have been defined based on the expression of the neoblast marker *Smedwi-1* and of specific transcription factors whose silencing impairs the regeneration of different cell types (Scimone et al., 2014). Thus, for example, a *FoxA* homolog is expressed in differentiated pharynx cells (*Smedwi-1* negative) and in *Smedwi-1*-positive cells in the mesenchyme surrounding this organ. These *FoxA/Smedwi-1* cells are lineage-specific progenitors; RNAi silencing of *FoxA* inhibits the differentiation of a new pharynx during regeneration (Adler et al., 2014). As described for pharyngeal progenitors, a collection of other transcription factors define different lineages, such as those that give rise to photoreceptors (Lapan and Reddien, 2011, 2012), protonephridia (Scimone et al., 2011), and several neuronal subpopulations (Cowles et al., 2013; Currie and Pearson, 2013; März et al., 2013; Scimone et al., 2014). A *myoD* homolog has been identified in the muscle lineage in *Schmidtea mediterranea* (Cebrià, 2000). *myoD* belongs to a family of well-known and evolutionarily conserved bHLH transcription factors that plays a key role in the commitment and differentiation of the skeletal myogenic lineage (Davis et al., 1987; Weintraub et al., 1991; Buckingham and Rigby, 2014). In planarians *myoD* is expressed in discrete subepidermal cells throughout the animal (especially on the ventral surface) that correspond to the body-wall musculature (Cebrià, 2000; Reuter et al., 2015), suggesting that planarian muscle is primarily skeletal in nature. *myoD* is also expressed in neoblasts, strongly suggesting that it is expressed in myogenic progenitors (Scimone et al., 2014). However, additional functional data is necessary to fully determine the role of *myoD* in planarian muscle differentiation. To date, the only relevant data indicate that planarians can regenerate after RNAi silencing of *myoD*, but form pointed blastemas and heads (Reddien et al., 2005b), possibly due to defects in the body-wall musculature.

### Early muscle differentiation during blastema formation

Previous studies based on morphological criteria and electron microscopy suggested that the first myogenic cells within the blastema are detectable on days 2–3 of regeneration (Sauzin, 1967; Pedersen, 1972; Hori, 1983; Morita and Best, 1984). More recently however, the use of the planarian monoclonal antibody TMUS-13 (Romero et al., 1991; Bueno et al., 1997a) against myosin heavy chain (MHC) clearly demonstrated that differentiating myocytes are present as early as day 1 of regeneration in a narrow strip of pre-existing tissue adjacent to the site of amputation (Cebrià et al., 1997). As regeneration proceeds, these myocytes also appear within the blastema, although some appear to intercalate with the pre-existing musculature outside the blastema (Cebrià et al., 1997). Myocytes at different stages of differentiation are observed within the blastema (Cebrià et al., 1997).

In systems in which regeneration involves the formation of a blastema within which the missing structures are formed, 2 main scenarios are proposed regarding the cellular nature of the blastema: (i) blastema cells are naïve undifferentiated cells that are committed and differentiate within the blastema; or (ii) blastema cells are a heterogeneous population of cells which enter the blastema already committed to specific cell lineages. Recent data from different models favor the latter scenario (Tanaka and Reddien, 2011; Reddien, 2013). Multiple studies characterizing the spatial and temporal distribution of distinct lineage-committed cell populations in planarians support this specialized progenitor model (Reddien, 2013; Scimone et al., 2014). Furthermore, this view is in agreement with ultrastructural observations indicating that neoblasts with a clear undifferentiated morphology are usually detected outside of the blastema, while those within the blastema show signs of differentiation (Morita et al., 1969; Pedersen, 1972; Hori, 1992). Morita et al. (1969) and Pedersen (1972) also described small groups or clusters of neoblasts with no morphological signs of differentiation in the boundary separating the blastema from the rest of the animal. Studies of the body-wall musculature using immunostaining with the TMUS-13 antibody against MHC and *in situ* hybridization for the *myoD* homolog also support this model: the first differentiating cells expressing these markers are seen at very early stages of regeneration in the pre-existing tissues adjacent to the blastema (Cebrià et al., 1997; Cebrià, 2000). As regeneration proceeds these myocytes migrate into the blastema where they fully differentiate to regrow the body-wall musculature (Cebrià et al., 1997).

### Pharyngeal muscle regeneration

**Figure 3:**
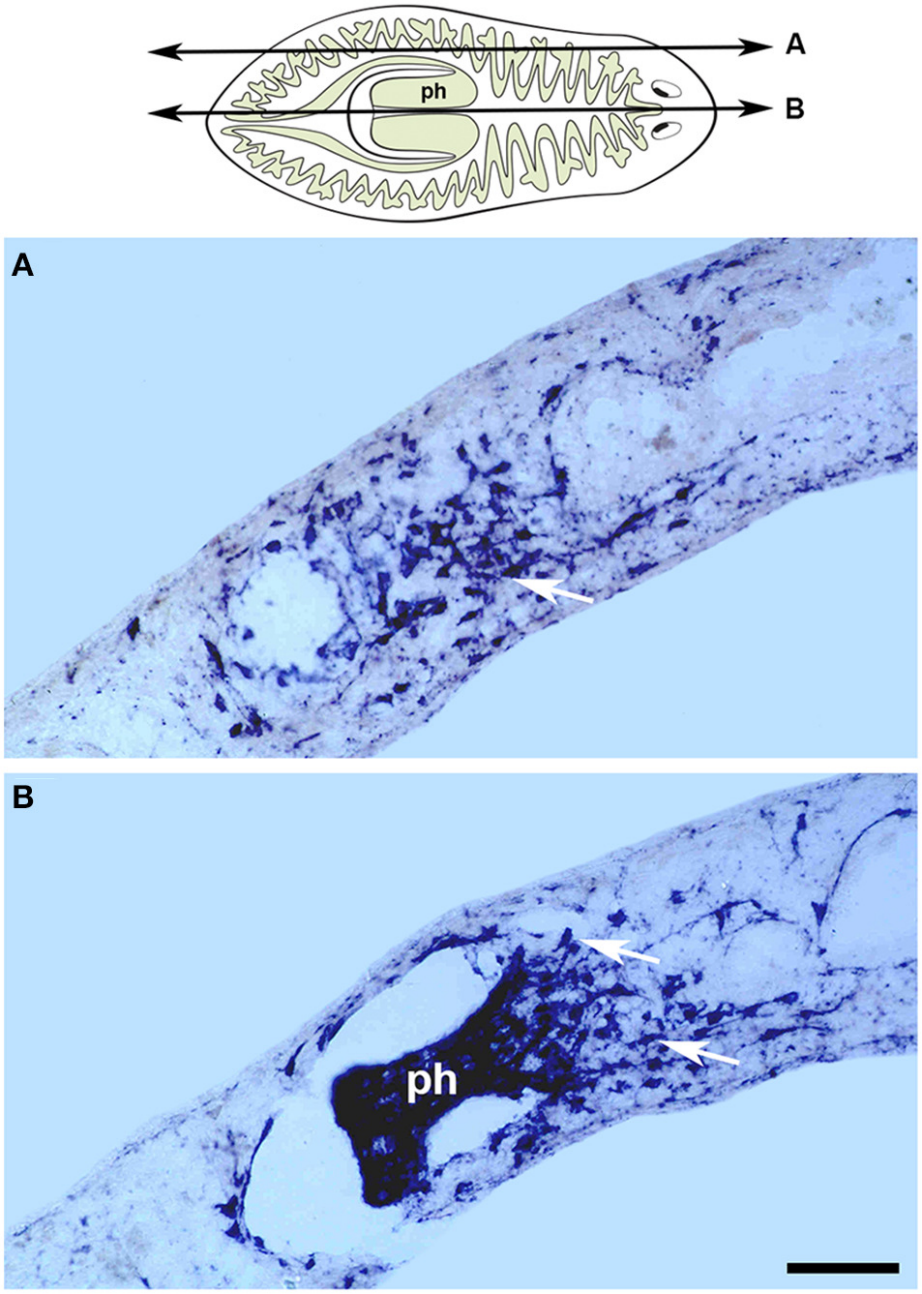
*****Gtmhc*** expression in the regenerating pharynx**. The cartoon represents the level and orientiation of the sagittal sections in **(A,B)**. *In situ* hybridizations for a *myosin heavy chain* gene on sagittal sections of regenerating tails from the species *Girardia tigrina*, 6 days after amputation. **(A)** Lateral section showing an accumulation of myocytes (arrow) in the mesenchyme surrounding the pharynx. **(B)** Central section containing the regenerated pharynx (ph) with myocytes evident in the mesenchyme at its base (arrows). Scale bar: 50 μm. Image adapted from Cebrià (2000). Anterior to the right. Dorsal to the top.

The planarian pharynx is a muscular tube delimited by external and internal monostratified epithelia. Circular and longitudinal fibers are found beneath these 2 epithelia, which are also connected by radial muscle fibers (Bueno et al., 1997a,b). This organ does not contain neoblasts and therefore pharynx regeneration and cell renewal depends on the entry of neoblasts from the mesenchyme. *In situ* hybridization for the *mhc* gene has shown that very early during the regeneration of new pharyngeal muscle, small *mhc-*expressing cells appear as early as days 1–2 in the mesenchymal space in a region defining the pharynx rudiment (Kobayashi et al., 1999). Importantly, throughout the entire process by which this rudiment grows into a new pharynx, *mhc-*expressing cells are consistently detected in the mesenchyme surrounding the pharynx rudiment as well as inside the rudiment itself (Kobayashi et al., 1999; Cebrià, 2000; Figure 3). These results suggest that during regeneration the new pharyngeal muscle cells are derived from the neoblasts in the mesenchymal space and migrate into the pharynx rudiment as pre-committed muscle progenitors. This scenario resembles that described above for the body-wall musculature, albeit with one important difference: whereas the myocytes detected outside the blastema during body-wall muscle regeneration are already positive for MHC protein (Cebrià et al., 1997), those surrounding the pharynx rudiment express the *mhc* gene but are not positive for MHC protein (Bueno et al., 1997b). The first myocytes expressing MHC are only detected within the pharynx rudiment at 5–6 days of regeneration (Bueno et al., 1997b), suggesting that regulation of MHC protein production may depend on different spatial and/or temporal cues in these 2 muscle types. It should be noted that although the TMUS-13 antibody recognizes all planarian muscle, the *mhc* genes expressed in the body-wall and pharynx are distinct.

In conclusion, more detailed studies are required to confirm these observations and unambiguously trace the origin of the new muscle progenitors, their migration, and ultimate fates inside the blastema, and to elucidate the exact role of *myoD* in planarian muscle determination and differentiation.

## Restoration of the body-wall muscle pattern

**Figure 4:**
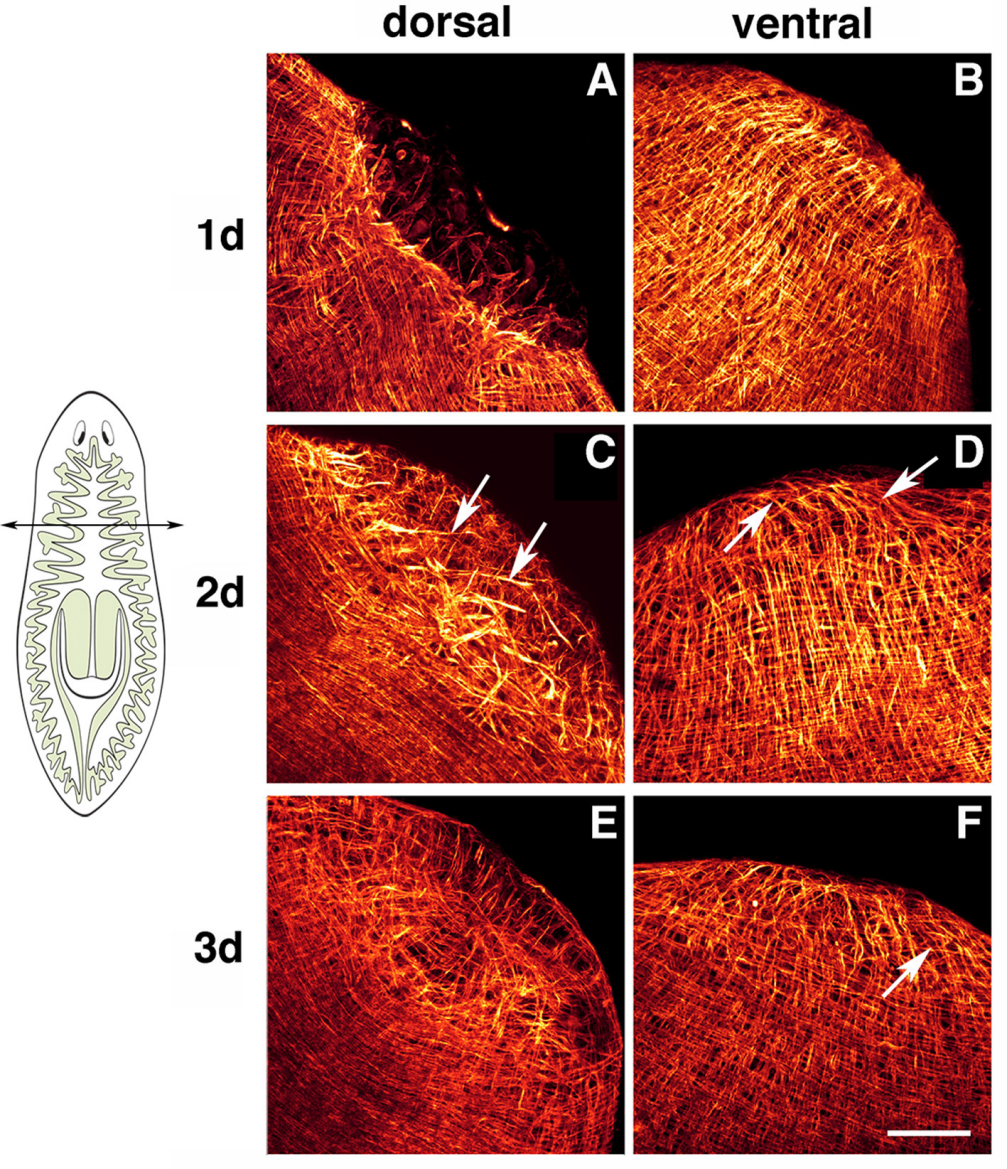
**Planarian body-wall muscle regeneration**. Whole-mount immunostaining with TMUS-13 antibody during head regeneration. Planarians were amputated at a pre-pharyngeal level as indicated in the cartoon. Head regeneration from the trunk piece was monitored. **(A,B)** Show dorsal and ventral views, respectively, of 1-day regenerants. A dorsal “hole” lacking fibers is evident in **(A)**. **(C,D)** Show dorsal and ventral views, respectively, of 2-day regenerants. Pre-existing dorsal longitudinal fibers enter the blastema (arrows in **C**). The ventral region of the blastema contains mainly longitudinal fibers (arrows in **D**). **(E,F)** Show dorsal and ventral views, respectively, of 3-day regenerants. Dorsally, the muscle fibers converge at the center of the blastema, restoring the pattern observed in intact planarians. Ventrally, new circular muscle fibers are evident (arrow). Scale bar: 50 μm. Image adapted from Cebrià and Romero (2001).

During regeneration, the intricate muscle fiber pattern of the body-wall musculature is not only fully restored, but also becomes a perfect extension of the pre-existing musculature. How this occurs remains unclear. As described above (Figure 1) the muscle pattern at the tip of the head, especially that of the longitudinal fibers, differs between the dorsal and ventral surfaces. During anterior regeneration morphological differences are also observed between dorsal and ventral sides of the blastema (Cebrià and Romero, 2001). Thus, at day 1 a “hole” lacking muscle fibers and delimited by disorganized pre-existing fibers is evident in the anterior-most part of the dorsal region (Figure 4A). By contrast, the muscle fibers of the ventral surface show a much more organized pattern, with longitudinal fibers running in parallel up to the anterior-most tip (Figure 4B), as also observed in intact heads. By day 2, pre-existing longitudinal fibers appear to elongate into the blastema, which retains a disorganized pattern (Figure 4C, arrows). At this stage, ventral fibers are not observed within the blastema. By day 3, the dorsal muscle fibers show an incipient arrangement resembling the pattern observed in intact animals, with longitudinal fibers converging centrally. New circular fibers are observed in the ventral region of the blastema (Figures 4E,F). In the following days the muscle pattern is completely restored (Cebrià and Romero, 2001).

It remains unclear whether the differences observed in the pattern and dynamics of the early regeneration of dorsal and ventral fibers are related to wound healing. Chandebois (1976, 1980) suggested that during anterior regeneration the dorsal epithelium expands to heal the wound whereas in posterior regenerant blastema the ventral epithelial cell population expands to heal the wound. Interestingly, during posterior regeneration in the flatworm *Macrostomum spp*. the wound appears to shift ventrally, resulting in an opposing dynamic to that described for anterior regeneration in planarians, in which the dorsal pre-existing fibers reach the caudal-most end of the posterior tip and a ventral “hole” containing very few muscle fibers is observed (Salvenmoser et al., 2001). Therefore, the dynamics of body-wall muscle pattern restoration may differ in anterior versus posterior regeneration. Further studies will be necessary to determine whether these differences are observed within the same animal, as to date anterior regeneration has been described in the planarian *Schmidtea mediterranea* and posterior regeneration in *Macrostomum spp*. If these differences do indeed exist it would be of interest to analyze in detail whether the manner in which wound healing occurs in anterior and posterior regions plays a role in determining polarity during regeneration, as proposed by Chandebois (1976, 1980).

Finally, it seems clear that in addition to the differentiating myocytes derived from the stump, pre-existing muscle fibers are always found within the blastema during regeneration (Cebrià et al., 1997). Longitudinal fibers appear to grow from the truncated pre-existing fibers, while the circular fibers appear *de novo* within the blastema (Cebrià and Romero, 2001). These pre-existing fibers may play a role in guiding the entering myocytes and/or mediating their arrangement in order to restore muscle pattern. For example, in the flatworm *Macrostomum spp*., new circular fibers develop from myocytes oriented perpendicularly to the longitudinal fibers (Salvenmoser et al., 2001). This predicted instructive/guiding role of the pre-existing muscle fibers for the restoration of the planarian body-wall musculature pattern would be in agreement with several studies in other models in which muscle founders cells or muscle cells serve also as a template or cues for the subsequent development and patterning of the musculature and other cell types (Ho et al., 1983; Jellies and Kristan, 1991; Farrell et al., 1996; Lee et al., 2013).

## Muscle cells may provide positional information during regeneration

During regeneration in planarians (and during daily cell turnover in uncut animals), pluripotent neoblasts must differentiate into all missing cell types. This process needs to be tightly regulated to ensure differentiation into the exact cells types required in each territory. As many cell types and organs are differentially distributed along the anteroposterior (AP) and dorsoventral (DV) axes, neoblasts need to receive precise information about the specific tissues that are missing in the different regenerative contexts (i.e., anterior vs. posterior regeneration). Little is known about how positional identities are maintained and re-established during planarian regeneration. Some findings suggest that such positional information resides in differentiated cells (Kato et al., 2001). Studies of the participation of the Wnt/β-catenin, BMP, and FGFR signaling pathways in axial polarity and patterning have identified a collection of genes with key roles in these events (Cebrià et al., 2002; Kobayashi et al., 2007; Molina et al., 2007, 2011; Orii and Watanabe, 2007; Reddien et al., 2007; Gurley et al., 2008; Iglesias et al., 2008; Petersen and Reddien, 2008, 2011; Felix and Aboobaker, 2010; Gaviño and Reddien, 2011). Because these genes show a regionalized expression along the body axes and their silencing by RNAi results in polarity and patterning defects they are collectively described as “position control genes” (PCGs) (Reddien, 2011). Remarkably, most of these genes display subepidermal expression and very often co-localize (Witchley et al., 2013). More interestingly, PCGs are co-expressed in 95.7–99.8% of all muscle cells analyzed from different body regions, including body-wall, enteric, and pharyngeal muscle cells (Witchley et al., 2013). During regeneration the polarity determinants *notum* (Petersen and Reddien, 2011) and *Wnt1* (Adell et al., 2009; Petersen and Reddien, 2009) are rapidly induced in muscle cells. This induction occurs in differentiated pre-existing muscle cells, dynamically altering the expression of PCGs in these cells in response to amputation. Moreover, planarian muscle cells can re-adjust the PCG expression profile to correspond to the area of the body axis in which they are positioned after amputation (Witchley et al., 2013; Reuter et al., 2015). Based on these observations a model has been proposed whereby planarian muscle cells, by expressing different combinations of PCGs, provide positional information to the surrounding neoblasts, which in turn differentiate into the required tissues and organs in response to specific cues arriving along the body axes (Witchley et al., 2013).

It will be interesting to test this model in future studies, for example by characterizing differences in PCG expression in specific muscle cells from different regions in various regenerative contexts, or by analyzing the regenerative capabilities of muscle-deficient planarians, if possible.

## Conclusions and perspectives

Freshwater planarians have a complex body-wall musculature that functions mainly as a skeletal support and during locomotion. In addition, the digestive system consists of a highly muscular tubular organ, the pharynx, and a highly ramified gut surrounded by enteric musculature that mediates peristaltic movements during food intake and digestion. At the gene expression level the 2 muscle types express distinct forms of myosin heavy-chain genes. Planarians can regenerate and renew any type of cell and tissue through the differentiation of neoblasts, a population of adult pluripotent stem cells. These animals thus constitute an ideal model for the *in vivo* study of how these stem cells become committed and differentiate into the muscle lineage during regeneration. Preliminary results suggest that neoblasts become committed to the myogenic lineage before they enter the regenerative blastema, supporting the recently proposed existence of specialized neoblasts. However, definitive experimental proof is required, preferably obtained by studying the distribution and dynamics of *myoD*-positive cells during regeneration. Similarly, the process by which new muscle fibers are integrated into the pre-existing muscle and the role of these pre-existing fibers as a scaffold during the regenerative process needs to be better characterized. Planarians are also useful for the study of the behavior of stem cells and muscle progenitors as animal's age, a line of investigation that may help explain the loss of muscle stem cells observed in aging mammals (Sousa-Victor et al., 2015). Finally, recent studies have suggested that planarian muscle cells may provide positional information to stem cells, thus regulating their fates.

Therefore, future studies should address some of the important lacuna that we have in the field mostly related to the regulation of the planarian stem cells and muscle progenitors. How conserved is the myogenic program in planarian stem cells compared to other systems? Is planarian *myoD* sufficient for the differentiation of the myogenic lineage? What about other important transcription factors such as *Mef2* genes and *pax7* required for myogenesis in other models? Also, the fact that mature muscle cells could provide with positional information to planarian stem cells may be of great relevance to understand the regulation of those stem cells, as the existence of a niche for their activity has not been shown so far in these animals.

Finally, recent studies have shown that, in mammals, stem cells may have different behaviors under homeostatic or regenerative scenarios meaning that different signals from their changing environments trigger specific behaviors in each situation (Donati and Watt, 2015). In this sense, planarians are an excellent model in which to study *in vivo* how stem cells and muscle progenitors could be differently regulated in these two scenarios (Adler and Sánchez Alvarado, 2015) and to determine what are the signals that induce the myogenic lineage in them. It has been suggested that differentiating signals for the neoblasts probably come from differentiated tissues (Adler and Sánchez Alvarado, 2015) but, where do you they come from for the myogenic lineage?

In conclusion, planarian muscle may represent an attractive paradigm in which to study basic aspects of regeneration including stem cell biology, pattern formation, and positional information with further implications for the field of regenerative medicine.

## Author contributions

The author confirms being the sole contributor of this work and approved it for publication.

### Conflict of interest statement

The author declares that the research was conducted in the absence of any commercial or financial relationships that could be construed as a potential conflict of interest.

## References

[B1] Adell T. Cebrià F. Saló E. (2014). Planarian totipotent stem cells, in Stem Cells: From Basic Research to Therapy, Vol. 1, Basic Stem Cell Biology, Tissue Formation during Development, and Model Organisms, ed Calegari F. Waskow C. (Boca Raton, FL: CRC Press), 433–472.

[B2] Adell T. Saló E. Boutros M. Bartscherer K. (2009). *Smed-Evi/Wntless* is required for beta-catenin-dependent and –independent processes during planarian regeneration. Development 136, 905–910. 10.1242/dev.033761 19211673

[B3] Adler C. E. Sánchez Alvarado A. (2015). Types or states? Cellular dynamics and regenerative potential. Trends Cell Biol. 25, 687–696. 10.1016/j.tcb.2015.07.008 26437587 PMC4628888

[B4] Adler C. E. Seidel C. W. McKinney S. A. Sánchez Alvarado A. (2014). Selective amputation of the pharynx identifies a FoxA-dependent regeneration program in planaria. eLife 3:e02238. 10.7554/eLife.02238 24737865 PMC3985184

[B5] Atala A. Lanza R. Thomson J. A. Nerem R. (2011). Principles of Regenerative Medicine, 2nd Edn. London: Academic Press.

[B6] Baguñà J. (2012). The planarian neoblast: the rambling history of its origin and some current black boxes. Int. J. Dev. Biol. 56, 19–37. 10.1387/ijdb.113463jb 22252540

[B7] Baguñà J. Romero R. (1981). Quantitative analysis of cell types during growth, degrowth and regeneration in the planarians *Dugesia mediterranea* and *Dugesia tigrina*. Hydrobiologia 84, 181–194.

[B8] Bárány M. (1967). ATPase activity of myosin correlated with speed of muscle shortening. J. Gen. Physiol. 50, 197–218. 10.1085/jgp.50.6.197 4227924 PMC2225740

[B9] Buckingham M. Rigby P. W. J. (2014). Gene regulatory networks and transcriptional mechanisms that control myogenesis. Dev. Cell 28, 225–238. 10.1016/j.devcel.2013.12.020 24525185

[B10] Bueno D. Baguñà J. Romero R. (1997a). Cell-, tissue-, and position-specific monoclonal antibodies against the planarian *Dugesia (Girardia) tigrina*. Histochem. Cell. Biol. 107, 139–149. 9062799 10.1007/s004180050098

[B11] Bueno D. Espinosa L. Baguñà J. Romero R. (1997b). Planarian pharynx regeneration in regenerating tail fragments monitored with cell-specific monoclonal antibodies. Dev. Genes Evol. 206, 425–434. 10.1007/s004270050072 27747385

[B12] Cebrià F. (2000). Determination, Differentiation and Restitution of the Muscle Pattern During Regeneration and Cell Renewal in Freshwater Planarians. Ph.D. Thesis, University of Barcelona.

[B13] Cebrià F. Adell T. Saló E. (2010). Regenerative medicine: lessons from planarians, in Stem Cells, Regenerative Medicine and Cancer, ed Singh S. R. (Hauppauge, NY: Nova Science Publishers), 29–68.

[B14] Cebrià F. Bueno D. Reigada S. Romero R. (1999). Intercalary muscle cell renewal in planarian pharynx. Dev. Genes Evol. 209, 249–253. 10.1007/s004270050249 10079368

[B15] Cebrià F. Kobayashi C. Umesono Y. Nakazawa M. Mineta K. Ikeo K.. (2002). *nou-darake*, a novel gene related to FGF receptors is involved in restricting brain tissues to the head region of planarians. Nature 419, 620–624. 10.1038/nature01042 12374980

[B16] Cebrià F. Romero R. (2001). Body-wall muscle restoration dynamics are different in dorsal and ventral blastemas during planarian anterior regeneration. Belg. J. Zool. 131, 5–9.

[B17] Cebrià F. Vispo M. Newmark P. A. Bueno D. Romero R. (1997). Myocyte differentiation and body wall muscle regeneration in the planarian *Girardia tigrina*. Dev. Genes Evol. 207, 306–316. 10.1007/s004270050118 27747428

[B18] Chandebois R. (1976). Histogenesis and Morphogenesis in Plaanrian Regeneration. Monographs in Develop mental Biology, Vol. 11 Basel: Karger. 775318

[B19] Chandebois R. (1980). The dynamics of wound closure and its role in the programming of planarian regeneration. II- DCistalization. Dev. Growth Differ. 22, 693–704. 10.1111/j.1440-169X.1980.00693.x 37281333

[B20] Clark R. B. (1964). Dynamics in Metazoan Evolution. The Origin of the Coelom and Segments. Oxford: Clarendon Press.

[B21] Cowles M. W. Brown D. D. Nisperos S. V. Stanley B. N. Pearson B. J. Zayas R. M. (2013). Genome-wide analysis of the bHLH gene family in plaanrians identifies factors required for adult neurogenesis and neuronal regeneration. Development 140, 4691–4702. 10.1242/dev.098616 24173799

[B22] Crezée M. (1975). Monograph of the Solenofilomorphidae (Turbellaria: Acoela). Int. Rev. Ges. Hydrobiol. 60, 769–845. 10.1002/iroh.19750600604

[B23] Currie K. W. Pearson B. J. (2013). Transcription factors *lhx1/5-1* and *pitx* are required for the maintenance and regeneration of serotonergic neurons in planarians. Development 140, 3577–3588. 10.1242/dev.098590 23903188

[B24] Davis R. L. Weintraub H. Lassar A. B. (1987). Expression of a single transfected cDNA converts fibroblasts to myoblasts. Cell 51, 987–1000. 10.1016/0092-8674(87)90585-X 3690668

[B25] Donati G. Watt F. M. (2015). Stem cell heterogeneity and plasticity in epithelia. Cell Stem Cell 16, 465–476. 10.1016/j.stem.2015.04.014 25957902

[B26] Dumont N. A. Wang Y. X. Rudnicki M. A. (2015). Intrinsic and extrinsic mechanisms regulating satellite cell function. Development 142, 1572–1581. 10.1242/dev.114223 25922523 PMC4419274

[B27] Ehlers U. (1985). Das Phylogenetische System der Plathelminthes. Stuttgart: Gustav Fischer Verlag.

[B28] Farrell E. R. Fernandes J. Keshishian H. (1996). Muscle organizers in *Drosophila*: the role of persistent larval fibers in adult flight muscle development. Dev. Biol. 176, 220–229. 10.1006/dbio.1996.0129 8660863

[B29] Felix D. A. Aboobaker A. A. (2010). The TALE class homeobox gene Smed-prep defines the anterior compartment for head regeneration. PLoS Genet. 6:e1000915. 10.1371/journal.pgen.1000915 20422023 PMC2858555

[B30] Gaviño M. A. Reddien P. W. (2011). A Bmp/Admp regulatory circuit controls maintenance and regeneration of dorsoventral polarity in planarians. Curr. Biol. 21, 294–299. 10.1016/j.cub.2011.01.017 21295483 PMC3079492

[B31] Gentile L. Cebrià F. Bartscherer K. (2011). The planarian flatworm: an *in vivo* model for stem cell biology and nervous system regeneration. Dis. Model Mech. 4, 12–19. 10.1242/dmm.006692 21135057 PMC3014342

[B32] Gurley K. A. Rink J. C. Sánchez Alvarado A. (2008). Beta-catenin defines head versus tail identity during planarian regeneration and homeostasis. Science 319, 323–327. 10.1126/science.1150029 18063757 PMC2755502

[B33] Hartman M. A. Spudich J. A. (2012). The myosin superfamily at a glance. J. Cell Sci. 125, 1627–1632. 10.1242/jcs.094300 22566666 PMC3346823

[B34] Ho R. K. Ball E. E. Goodman C. S. (1983). Muscle pioneers: large mesodermal cells that erect a scaffold for developing muscles and motoneurones in grasshopper embryos. Nature 301, 66–69. 10.1038/301066a0 6337338

[B35] Hooge M. D. (2001). Evolution of body-wall musculature in the Platyhelminthes (Acoelomorpha, Catenulida, Rhabditophora). J. Morphol. 249, 171–194. 10.1002/jmor.1048 11517463

[B36] Hori I. (1983). Differentiation of myoblasts in the regenerating planarian *Dugesia japonica*. Cell Differ. 12, 155–163. 10.1016/0045-6039(83)90005-2

[B37] Hori I. (1992). Cytological approach to morphogenesis in the planarian blastema. I. Cell behavior during blastema formation. J. Submicrosc. Cytol. Pathol. 24, 75–84. 9066147

[B38] Iglesias M. Gómez-Skarmeta J. L. Saló E. Adell T. (2008). Silencing of *Smed-betacatenin1* generates radial-like hypercephalized planarians. Development 135, 1215–1221. 10.1242/dev.020289 18287199

[B39] Jellies J. Kristan W. B. (1991). The oblique muscle organizer in *Hirudo medicinalis*, an identified embryonic cell projecting multiple parallel growth cones in an orderly array. Dev. Biol. 148, 334–354. 10.1016/0012-1606(91)90342-Z 1936570

[B40] Kato K. Orii H. Watanabe K. Agata K. (2001). Dorsal and ventral positional cues required for the onset of planarian regeneration may reside in differentiated cells. Dev. Biol. 233, 109–121. 10.1006/dbio.2001.0226 11319861

[B41] Kobayashi C. Kobayashi S. Orii H. Watanabe K. Agata K. (1998). Identification of two distinct muscles in the planarian *Dugesia japonica* by their expression of myosin heavy-chain genes. Zool. Sci. 15, 861–869. 10.2108/zsj.15.861

[B42] Kobayashi C. Saito Y. Ogawa K, Agata, K. (2007). Wnt signaling is required for antero-posterior patterning of the planarian brain. Dev. Biol. 306, 714–724. 10.1016/j.ydbio.2007.04.010 17498685

[B43] Kobayashi C. Watanabe K. Agata K. (1999). The process of pharynx regeneration in planarians. Dev. Biol. 211, 27–38. 10.1006/dbio.1999.9291 10373302

[B44] Lanzavecchia G. (1977). Morphological modulations in helical muscles (Aschelminthes and Annelida). Int. Rev. Cytol. 51, 133–186. 10.1016/S0074-7696(08)60227-2 338536

[B45] Lapan S. W. Reddien P. W. (2011). *dlx* and *sp6-9* control optic cup regeneration in a prototypic eye. PLoS Genet. 7:e1002226. 10.1371/journal.pgen.1002226 21852957 PMC3154955

[B46] Lapan S. W. Reddien P. W. (2012). Transcriptome analysis of the planarian eye identifies *ovo* as a specific regulator of eye regeneration. Cell Reports 2, 294–307. 10.1016/j.celrep.2012.06.018 22884275 PMC3785364

[B47] Lee A. S. Harris J. Bate M. Vijaraghavan K. Fisher L. Tajbakhsh S.. (2013). Initiation of primary myogenesis in amniote limb muscles. Dev. Dyn. 242, 1043–1055. 10.1002/dvdy.23998 23765941

[B48] Lepper C. Partridge T. A. Fan C. M. (2011). An absolute requirement for Pax7-positive satellite cells in acute injury-induced muscle regeneration. Development 138, 3639–3646. 10.1242/dev.067595 21828092 PMC3152922

[B49] MacRae E. K. (1963). Observations on the fine structure of the pharyngeal muscle in the planarian *Dugesia tigrina*. J. Cell Biol. 18, 651–662. 10.1083/jcb.18.3.651 14064114 PMC2106326

[B50] MacRae E. K. (1965). The fine structure of muscle in a marine turbellarian. Z. Zellforsch. 68, 348–362. 10.1007/BF00342552 5869601

[B51] März M. Seebeck F. Bartscherer K. (2013). A Pitx transcription factor controls the establishment and maintenance of the serotonergic lineage in planarians. Development 140, 4499–4509. 10.1242/dev.100081 24131630

[B52] Molina M. D. Neto A. Maeso I. Gómez-Skarmeta J. L. Saló E. Cebrià F. (2011). Noggin and noggin-like genes control dorsoventral axis regeneration in planarians. Curr. Biol. 21, 300–305. 10.1016/j.cub.2011.01.016 21295481

[B53] Molina M. D. Saló E. Cebrià F. (2007). The BMP pathway is essential for re-specification and maintenance of the dorsoventral axis in regenerating and intact planarians. Dev. Biol. 311, 79–94. 10.1016/j.ydbio.2007.08.019 17905225

[B54] Moraczewski J. (1981). The fine structure of some Catenulida (Turbellaria Archoophora). Zoomorphologie 88, 65–80. 10.1007/BF00993304

[B55] Morita M. (1965). Electron microscopic studies on planaria. I. Fine structure of muscle fiber in head of the planarian *Dugesia dorotocephala*. J. Ultrastruct. Res. 13, 383–395. 10.1016/S0022-5320(65)90002-X 5848837

[B56] Morita M. Best J. B. (1984). Electron microscopic studies of planarian regeneration. *III*. Degeneration and differentiation of muscles. J. Exp. Zool. 229, 413–424. 10.1002/jez.1402290309 4820343

[B57] Morita M. Best J. B. Noel J. (1969). Electron microscopic studies of planarian regeneration. *I*. Fine structure of neoblasts in *Dugesia dorotocephala*. J. Ultrastruct. Res. 27, 7–23. 10.1016/S0022-5320(69)90017-3 5769728

[B58] Newmark P. A. Sánchez Alvarado A. (2000). Bromodeoxyuridine specifically labels the regenerative stem cells of planarians. Dev. Biol. 220, 142–153. 10.1006/dbio.2000.9645 10753506

[B59] Newmark P. A. Sánchez Alvarado A. (2002). Not your father's planarian: a classic model enters the era of functional genomics. Nat. Rev. Genet. 3, 210–219. 10.1038/nrg759 11972158

[B60] Orii H. Ito H. Watanabe K. (2002). Anatomy of the planarian Dugesia japonica I. The muscular system revealed by antisera against myosin heavy chains. Zool. Sci. 19, 1123–1131. 10.2108/zsj.19.1123 12426474

[B61] Orii H. Watanabe K. (2007). Bone morphogenetic protein is required for dorsoventral patterning in the planarian *Dugesia japonica*. Dev. Growth Differ. 49, 345–349. 10.1111/j.1440-169X.2007.00931.x 17501910

[B62] Pedersen K. J. (1972). Studies on regeneration blastemas of the planarian *Dugesia tigrina* with special reference to differentiation of the muscle-connective tissue filament system. Wilhelm Roux's Arch. EntwMech. Org. 169, 134–169. 10.1007/BF00649889 28304777

[B63] Petersen C. P. Reddien P. W. (2008). *Smed-betacatenin-1* is required for anteroposterior blastema polarity in planarian regeneration. Science 319, 327–330. 10.1126/science.1149943 18063755

[B64] Petersen C. P. Reddien P. W. (2009). A wound-induced Wnt expression program controls planarian regeneration polarity. Proc. Natl. Acad. Sci. U.S.A. 106, 17061–17066. 10.1073/pnas.0906823106 19805089 PMC2743725

[B65] Petersen C. P. Reddien P. W. (2011). Polarized notum activation at wounds inhibits Wnt function to promote planarian head regeneration. Science 332, 852–855. 10.1126/science.1202143 21566195 PMC3320723

[B66] Prudhoe S. (1985). A Monograph on Polyclad Turbellaria. New York, NY: Oxford University Press.

[B67] Reddien P. W. (2011). Constitutive gene expression and the specification of tissue identity in adult planarian biology. Trends Genet. 27, 277–285. 10.1016/j.tig.2011.04.004 21680047 PMC3125669

[B68] Reddien P. W. (2013). Specialized progenitors and regeneration. Development 140, 951–957. 10.1242/dev.080499 23404104 PMC3583037

[B69] Reddien P. W. Bermange A. L. Kicza A. M. Sánchez Alvarado A. (2007). BMP signaling regulates the dorsal planarian midline and is needed for asymmetric regeneration. Development 134, 4043–4051. 10.1242/dev.007138 17942485

[B70] Reddien P. W. Bermange A. L. Murfitt K. J. Jennings J. R. Sánchez Alvarado A. (2005b). Identification of genes needed for regeneration, stem cell function, and tissue homeostasis by systematic gene perturbation in planaria. Dev. Cell 8, 635–649. 10.1016/j.devcel.2005.02.014 15866156 PMC2267917

[B71] Reddien P. W. Oviedo N. J. Jennings J. R. Jenkin J. C. Sánchez Alvarado A. (2005a). SMEDWI-2 id a PIWI-like protein that regulates planarian stem cells. Science 310, 1327–1330. 10.1126/science.1116110 16311336

[B72] Reddien P. W. Sánchez Alvarado A. (2004). Fundamentals of planarian regeneration. Annu. Rev. Cell Dev. Biol. 20, 725–757. 10.1146/annurev.cellbio.20.010403.095114 15473858

[B73] Reuter H. März M. Vogg M. C. Eccles D. Grifol-Boldú L. Wehner D.. (2015). β-catenin-dependent control of positional information along the AP body axis in planarians involves a *teashirt* family member. Cell Reports 10, 1–13. 10.1016/j.celrep.2014.12.018 25558068

[B74] Reuter M. (1977). Ultrastructure of the stylet protractor muscle in *Gyratrix hermaphrodites* (Turbellaria, Rhabdocoela). Acta Zool. 58, 179–184. 10.1111/j.1463-6395.1977.tb00253.x

[B75] Rieger R. M. Mainitz M. (1977). Comparative fine structure study of the body wall in gnathostomulids and their phylogenetic position between Pltyhelminthes and Aschelminthes. Z. Zool. Syst. Evolutionsforsch. 15, 9–35. 10.1111/j.1439-0469.1977.tb00530.x

[B76] Rieger R. M. Salvenmoser W. Legniti A. Tyler S. (1994). Phalloidin-rhodamine preparations of *Macrostomum hystricinum marinum* (Plathelminthes). Morphology and postembryonic development of the musculature. Zoomorphology 114, 133–147. 10.1007/BF00403261

[B77] Rieger R. M. Tyler S. Smith J. P. S. III. Rieger G. E. (1991). Platyhelminthes: Turbellaria. A Microscopic Anatomy of Invertebrates, Vol. 3: Platyhelminthes and Nemertinea. New York, NY: Wiley-Liss.

[B78] Rink J. C. (2013). Stem cell systems and regeneration in planaria. Dev. Genes Evol. 223, 67–84. 10.1007/s00427-012-0426-4 23138344 PMC3552358

[B79] Riser N. W. (1987). *Nemertinoides elongatus* gen.n.sp.n. (Turbellaria: Nemertodermatida) from coarse sand beaches of the western North Atlantic. Proc. Helminthol. Soc. Wash. 54, 60–67.

[B80] Romero R. Fibla J. Bueno D. Sumoy L. Soriano M. A. Baguñà J. (1991). Monoclonal antibodies as markers of specific cell types and regional antigens in the freshwater planarian *Dugesia (G.) tigrina*. Hydrobiologia 227, 73–79.

[B81] Salvenmoser W. Riedl D. Ladurner P. Rieger R. (2001). Early steps in the regeneration of the musculature in *Macrostomum* sp. (Macrostomorpha). Belg. J. Zool. 131, 105–109.

[B82] Sambasivan R. Yao R. Kissenpfennig A. Van Wittenberghe L. Paldi A. Gayraud-Morel B.. (2011). Pax7-expressing satellite cells are indispensable for adult skeletal muscle regeneration. Development 138, 3647–3656. 10.1242/dev.067587 21828093

[B83] Sandoval-Guzmán T. Wang H. Khattak S. Schuez M. Roensch K. Nacu E.. (2014). Fundamental differences in dedifferentiation and stem cell recruitment during skeletal muscle regeneration in two salamander species. Cell Stem Cell 14, 174–187. 10.1016/j.stem.2013.11.007 24268695

[B84] Sarnat H. B. (1984). Muscle histochemistry of the planarian *Dugesia tigrina* (Turbellaria: Tricladida): implications in the evolution of muscle. Trans. Am. Microsc. Soc. 103, 284–294. 10.2307/3226190

[B85] Sauzin M. J. (1967). Étude ultrastructurale de la différenciation au cours de la régénération de la planaire *Dugesia gonocephala*. II. Différenciation musculaire. Bull. Soc. Zool. Fr. 92, 613–619.

[B86] Scimone M. L. Kravarik K. M. Lapan S. W. Reddien P. W. (2014). Neoblast specialization in regeneration of the planarian *Schmidtea mediterranea*. Stem Cell Rep. 3, 339–352. 10.1016/j.stemcr.2014.06.001 25254346 PMC4176530

[B87] Scimone M. L. Srivastava M. Bell G. W. Reddien P. W. (2011). A regulatory program for excretory system regeneration in planarians. Development 138, 4387–4398. 10.1242/dev.068098 21937596 PMC3177309

[B88] Sousa-Victor P. García-Prat L. Serrano A. L. Perdiguero E. Muñoz-Cánoves P. (2015). Muscle stem cell aging: regeneration and rejuvenation. Trends Endocrinol. Metab. 26, 287–296. 10.1016/j.tem.2015.03.006 25869211

[B89] Tanaka E. M Reddien P. W. (2011). The cellular basis for animal regeneration. Dev. Cell 21, 172–185. 10.1016/j.devcel.2011.06.016 21763617 PMC3139400

[B90] Wagner D. E. Wang I. E. Reddien P. W. (2011). Clonogenic neoblasts are pluripotent adult stem cells that underlie planarian regeneration. Science 332, 811–816. 10.1126/science.1203983 21566185 PMC3338249

[B91] Weintraub H. Davis R. Tapscott S. Thayer M. Krause M. Benezra R.. (1991). The myoD gene family: nodal point during specification of the muscle cell lineage. Science 251, 761–766. 10.1126/science.1846704 1846704

[B92] Westblad E. (1949). On Meara stichopi (Bock) Westblad, a new representative of Turbellaria Archoophora. Ark. Zool. Ser. 21, 43–57.

[B93] Witchley J. N. Mayer M. Wagner D. E. Owen J. H. Reddien P. W. (2013). Muscle cells provide instructions for planarian regeneration. Cell Rep. 4, 1–9. 10.1016/j.celrep.2013.07.022 23954785 PMC4101538

